# Journal of Applied Clinical Medical Physics Editorial

**DOI:** 10.1120/jacmp.v5i3.2066

**Published:** 2004-10-21

I want to report that the launch of our double‐blind procedure for manuscript review has been successful, thanks to the diligence and discretion of our Associate Editors and Reviewers. Our goal was to publish four issues in the calendar year 2004. We are going to meet this goal: the Fall issue is scheduled for publication in December. I wish to offer many thanks to all of you. You have all shown faith in the JACMP and supported it.

I also want to recognize the many others who make the JACMP possible. The JACMP survives on volunteer efforts. Everyone associated with the JACMP volunteers his or her time and efforts. These individuals, like you, are deeply committed to and firmly believe in the need for a clinically oriented journal. Below you will see listed the names of many of these volunteers. When you see them in person, I would greatly appreciate it if you let them know how much you appreciate their efforts on behalf of the JACMP.

In addition, and as mentioned in the previous issue, we owe a tremendous debt of gratitude to our commercial sponsors. You can show your gratitude in a number of ways. For instance, please keep in mind we are counting every use of our banner ads. Every time you visit a vendor through the JACMP, you help the JACMP. Using the banner ads demonstrates to our sponsors the importance of the journal to you and the effectiveness of our journal as an avenue to reach them. Another way to show your thanks is to consider our sponsors whenever you need a product or service or want to visit a vendor's web site: do so using the banner ads. Use the list of our sponsors. Mention the journal in your communications with them. Ultimately, our existence depends on you in so many ways, reading our articles, recognizing our volunteers, and supporting our sponsors. We hope you continue to make the JACMP and its support an easy habit.

Michael D. Mills

Editor‐in‐Chief

**Table:** 

**The Journal of Applied Clinical Medical Physics**	
**Editorial Board 2004**	
Editor‐in‐Chief	Michael D. Mills
Deputy Editor‐in‐Chief	Timothy D. Solberg
Associate Editor‐at‐Large	Richard Stark
**Associate Editors**	
Radiation Oncology Physics	Nzhde Agazaryan, B. Gino Fallone
	John Gibbons, Michael Herman
	Edwin McCullough, Timothy Paul
	Matthew Podgorsak
	Nikos Papanikolaou
Medical Imaging	Walter Huda, William Pavlicek
Radiation Measurements	Larry DeWerd
Radiation Protection and Regulations	James Deye
Nonionizing Topics	Bhudatt Paliwal
Other Topics	Timothy Solberg
Book/Media Reviews	James Smathers
Editorials	John Horton
Journal Production Team	
Manuscript Manager	Patricia Walker
Copy Editor	Betty R. Robinson
Layout Editor	Frances Robinson
Proofreader	Heather Shand

